# Mitochondrial Genetic Drift after Nuclear Transfer in Oocytes

**DOI:** 10.3390/ijms21165880

**Published:** 2020-08-16

**Authors:** Mitsutoshi Yamada, Kazuhiro Akashi, Reina Ooka, Kenji Miyado, Hidenori Akutsu

**Affiliations:** 1 Department of Obstetrics and Gynecology, Keio University School of Medicine, 35 Shinanomachi Shinjuku-ku, Tokyo 160-8582, Japan; akashikazu@gmail.com (K.A.); reinaooka@gmail.com (R.O.); 2 Department of Reproductive Biology, National Research Institute for Child Health and Development, 2-10-1 Okura Setagaya-ku, Tokyo 157-8535, Japan; miyado-k@ncchd.go.jp (K.M.); akutsu-h@ncchd.go.jp (H.A.)

**Keywords:** Mitochondria DNA (mtDNA), nuclear transfer, mitochondria replacement (MR), nDNA–mtDNA compatibility, mtDNA–mtDNA compatibility, mtDNA replicative segregation, mitochondrial function, mtDNA genetic drift, maternal inheritance, mtDNA heteroplasmy

## Abstract

Mitochondria are energy-producing intracellular organelles containing their own genetic material in the form of mitochondrial DNA (mtDNA), which codes for proteins and RNAs essential for mitochondrial function. Some mtDNA mutations can cause mitochondria-related diseases. Mitochondrial diseases are a heterogeneous group of inherited disorders with no cure, in which mutated mtDNA is passed from mothers to offspring via maternal egg cytoplasm. Mitochondrial replacement (MR) is a genome transfer technology in which mtDNA carrying disease-related mutations is replaced by presumably disease-free mtDNA. This therapy aims at preventing the transmission of known disease-causing mitochondria to the next generation. Here, a proof of concept for the specific removal or editing of mtDNA disease-related mutations by genome editing is introduced. Although the amount of mtDNA carryover introduced into human oocytes during nuclear transfer is low, the safety of mtDNA heteroplasmy remains a concern. This is particularly true regarding donor-recipient mtDNA mismatch (mtDNA–mtDNA), mtDNA-nuclear DNA (nDNA) mismatch caused by mixing recipient nDNA with donor mtDNA, and mtDNA replicative segregation. These conditions can lead to mtDNA genetic drift and reversion to the original genotype. In this review, we address the current state of knowledge regarding nuclear transplantation for preventing the inheritance of mitochondrial diseases.

## 1. Introduction

Mitochondria are eukaryotic organelles that play an essential role in energy production [1]. Mitochondrial function and replication are regulated by both mitochondrial and nuclear genomes. Human mitochondrial DNA (mtDNA) consists of a double-stranded circular DNA molecule of 16,569 base pairs (bp), containing 37 genes coding for 13 subunits of the 83 enzymes involved in oxidative phosphorylation, 22 tRNAs, and 2 rRNAs required for translating the transcripts of mitochondrial-encoded polypeptides. Mitochondria contain more than 1000 different proteins, most of which are encoded in nDNA and therefore require targeting to and import by the mitochondria [2]. Although there are exceptional reports that paternal mtDNA can be passed to offspring [3], usually sperm mitochondria are ubiquitinated inside the oocyte cytoplasm and are later subjected to proteolysis during preimplantation development [4]. Therefore, mtDNA is almost exclusively maternally inherited by transmission from the mother via the oocyte cytoplasm.

Defective mitochondria can cause mtDNA diseases, including mitochondrial encephalomyopathy, lactic acidosis, stroke-like episodes (MELAS), myoclonus epilepsy associated with ragged-red fibers, neurogenic muscle weakness, ataxia, retinitis pigmentosa, Leber hereditary optic neuropathy (LHON), Leigh’s syndrome, diabetes, and deafness. Approximately 1 in 5,000 births carry a pathogenic mtDNA mutation [5]. In fact, mtDNA mutations are common; a survey of newborn cord blood showed that 1 out of 200 infants had one of the ten most common pathogenic mtDNA mutations [6]. It is also known that with aging, the number of mitochondrial deletions and point mutations increases significantly, and that up to 50% of human oocytes contain mutated or deleted mtDNA sequences [7,8].

**Figure 1:**
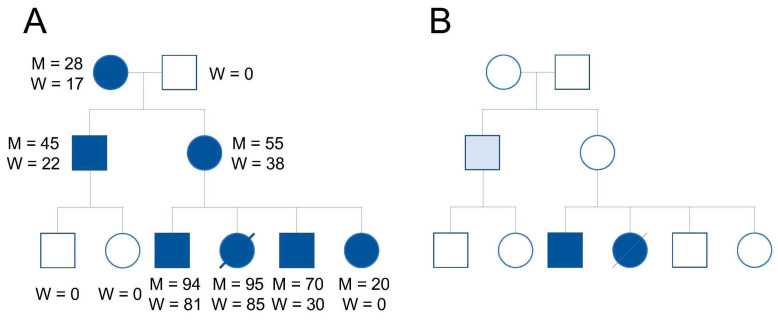
Mitochondrial and clinical phenotype correlations in disorders associated with mtDNA mutations. (**A**) All maternal relatives harbor a mutated mtDNA (dark symbols); M indicates the mtDNA heteroplasmy ratio in muscle; W indicates the mtDNA heteroplasmy ratio in white blood cells. (**B**) Clinical phenotype is observed only when the mtDNA heteroplasmy ratio reaches a certain threshold (dark symbols). The pedigree image was adapted from DiMauro and Moraes [11].

Unlike nDNA, which contains only two copies of each gene per cell, there are multiple copies of mtDNA in each eukaryotic cell [9]. Although mtDNA copy number differs by cell type, each cell can have a mixture of mitochondria with different degrees of mutation; a phenomenon referred to as heteroplasmy. In contrast, homoplasmy describes those cells that contain only one mtDNA variant. The proportion of mtDNA heteroplasmy can shift during both meiotic and mitotic cell division, a process known as replicative segregation (reviewed in [10]) (Figure 1). However, not all offspring inheriting a pathogenic mtDNA mutation will express a symptomatic phenotype because disease severity depends on the particular mutation, the proportion of normal to mutated mtDNA in each cell, and the energy requirements of each tissue. Clinical symptoms become visible when the level of mutated mtDNA exceeds a tissue-specific critical threshold, which is known as the threshold effect for phenotypic expression (reviewed in [5]). The effect of mtDNA heteroplasmy is striking in the mutation associated with MELAS; clinical phenotypes such as cardiomyopathy, migraines, diabetes mellitus, and deafness occur when the mutated mtDNA heteroplasmy rate is high. In a pedigree with a high prevalence of mtDNA heteroplasmy members, meiotic segregation of mutated mtDNAs can yield a wide range of phenotypes, from asymptomatic to lethal disease (reviewed in [10,11]) (Figure 1). Since mtDNA heteroplasmy may vary between tissues, the severity of damage will vary among organs from the same individual.

The current conventional approach for preventing mtDNA disease transmission is preimplantation genetic testing for monogenic disorders (PGT-M). PGT-M is an in vitro fertilization (IVF)-based technique in which the biopsy procedure of preimplantation of embryos consists of two main steps: (1) creating an opening in the zona pellucida and (2) removing polar bodies (PBs) or embryonic cells at cleavage and blastocyst stages [12]. These biopsied samples are subjected to genetic testing, enabling the selection of mutation-free or low mutation load embryos for transfer into the uterus. However, PGT-M has some diagnostic limitations. In the case of mtDNA mutations, embryonic mitochondria may shift their heteroplasmy levels during replicative segregation, which can compromise the reliability of the PGT-M. In addition, in the case of LHON, which is typically caused by mtDNA homoplasmy for mutated mtDNA, PGT-M is of little use for women carrying in their oocytes mtDNA homoplasmy for the mutated mtDNA.

## 2. Nuclear Transfer for Mitochondrial Replacement

**Figure 2:**
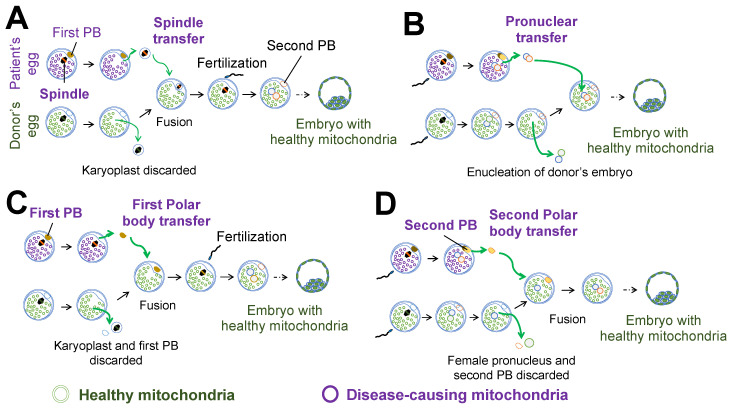
Nuclear transfer for mitochondria replacement. (**A**) Spindle transfer. (**B**) Pronuclear transfer. (**C**) First polar body transfer. (**D**) Second polar body transfer.

Nuclear transfer techniques such as maternal spindle transfer (MST), pronuclear transfer (PNT), first polar body transfer (PB1T), and second polar body transfer (PB2T) are all types of mitochondrial replacement therapy (MRT) in which a patient’s egg is used for transferring either a spindle isolated from an oocyte in maternal metaphase II (in MST) or the first polar body (in PB1T) into a donor’s egg (Figure 2A,C). Additional strategies use a patient’s egg for transferring into a donor’s zygote either a pronuclei isolated from a zygote (in PNT) or the second polar body (in PB2T) (Figure 2B,D). Each technique involves removing the genome from the recipient’s egg and subsequently transferring it to an enucleated oocyte or zygote provided by a healthy donor, which simultaneously replaces the recipient’s disease-causing mitochondria with disease-free mitochondria. Therefore, these techniques are expected to allow the transmission of nDNA from both parents, while reducing the risk of transmitting mutant mtDNA to their offspring. At present, there are no fundamental cures for mitochondrial diseases and most of the available treatments are aimed at relieving symptoms. Although PGT-M is a useful option to reduce the risk of abnormal mtDNA transmission, the exact threshold of the mtDNA heteroplasmy ratio is still arbitrary (reviewed in [10]). Therefore, new treatments for preventing mtDNA disease transmission to the next generation are needed.

## 3. Developmental Potential and Mitochondrial Carryover after Nuclear Transfer

The risk of transmitting deleterious mtDNA mutations from mother to child may be reduced by MRT by transferring nDNA from an oocyte carrying mutated mtDNA into an oocyte cytoplasm from a healthy donor containing, presumably, disease-free mitochondria. However, there is a concern that genome transfer may impair developmental potential and genotype. Accordingly, several animal studies have examined developmental potential and conducted genotype analysis following genome transfer.

The first reliable example of successful nuclear transfer in mammals was published in 1983 by McGrath and Solter [13]. They performed PNT between two different mouse strains; the reconstructed embryos transferred into the uterus successfully yielded litters. Transmitochondrial mouse (MitoMouse) zygotes, which have a massive deletion of mtDNA that causes respiratory disorders and leads to the development of mitochondrial disease, have also been used for PNT. In this case, both paternal and maternal pronuclei of the zygotes with pathogenic mtDNA mutations were transferred into enucleated healthy zygotes [14]. Although offspring derived from the reconstructed embryos were rescued from the development of respiratory defects throughout their lives, the progeny from the PNT harbored 6–21% (average 11%) mutant mtDNA. In human studies, Craven et al. performed PNT using abnormally fertilized embryos (unipronuclear or tripronuclear); the blastocyst development rate of fertilized eggs after PNT was half that of the fertilized eggs without genome transfer [15]. The amount of mtDNA carryover in embryos after PNT fluctuated 8.1% ± 7.6 (mean ± standard deviation). To improve developmental efficiency and reduce mtDNA carryover, a further study was performed to adjust the timing of the nuclear transfer to shortly after completion of meiosis, rather than shortly before the first mitotic division, using normal fertilized human embryos [16]. After optimization, the blastocyst development rate improved, and blastocyst formation and quality did not differ between non-manipulated controls and technical controls. mtDNA carryover in the majority of embryos was reduced to less than 2% by omitting sucrose from the manipulation medium; however, 21% of the embryos still exhibited a mtDNA heteroplasmy ratio greater than 5%.

To further reduce mtDNA carryover, genome transfer at developmental stages earlier than the pronuclear stage was attempted. In a study using macaque monkeys, MST produced healthy babies with no apparent adverse health effects over three years [17]. Although mtDNA carryover was very low, two of 24 oocytes derived from MST offspring contained a substantial degree (16.2% and 14.1%) of mtDNA heteroplasmy [18]. In humans, successful blastocyst development and establishment of embryonic stem cell (ESC) lines have been demonstrated by replacing maternal mtDNA with donor-derived mtDNA using MST [19,20]. Genome transfer using human metaphase II oocytes resulted in less than 0.5% mtDNA carryover in the majority of reconstructed oocytes. In addition, parthenogenetically activated oocytes efficiently developed to the blastocyst stage and isolated ESCs differentiated into neurons, cardiomyocytes, and pancreatic beta cells. Stem cells and differentiated cells showed normal mitochondrial function and maintained the new mitochondrial genotype for over a year [19]. Parthenogenesis can exclude sperm factors to study the effect of different mtDNA haplotypes derived from maternal and oocyte donors. Furthermore, to study nDNA–mtDNA mismatch, several human ESC lines composed of different nDNA–mtDNA combinations were isolated using MST and somatic cell nuclear transfer (SCNT) (Table 1). The developmental ratio, mtDNA genotype, and mitochondrial function were observed using ESCs and fibroblasts differentiated from these stem cell lines [21]. To improve clinical applications of MST in humans, cryopreserved oocytes were used for genome transfer, which facilitates synchronization of genome transfer between donor and recipient. Although vitrification of either nDNA or cytoplasm allowed development to the blastocyst stage and stem cell derivation, development was more efficient when cryopreserved nDNA and fresh recipient cytoplasm were used. This suggests that oocyte cytoplasm is susceptible to damage due to freezing and thawing. The blastocyst development rate and stem cell derivation efficiency were similar to the results observed in parthenogenesis, suggesting that manipulation did not impair development. Similar results were reported by Kang et al. [22]. In 2020, a mouse study showed that MST between B6CBAF1 and NZB lines resulted in healthy litters; mtDNA heteroplasmy was reduced in F2 mice and it was undetectable in subsequent generations [23].

## 4. mtDNA Genetic Drift after Mitochondria Replacement

Small amounts of mtDNA can be found in karyoplasts, accounting for less than 2% of the mtDNA content in reconstructed oocytes after nuclear transfer [16,19,22,24]. Even though low levels of heteroplasmy introduced into human oocytes by mitochondrial carryover during nuclear transfer often vanish, concerns remain regarding mtDNA genetic drift, which can cause a reversion to the maternal mtDNA due to incompatibility between maternal mtDNA and cytoplasmic donor-derived mtDNA. Sharpley et al. showed that the proportion of NZB mtDNA in NZB-129 heteroplasmic mice (NZB mtDNA and 129S6 mtDNA) was preferentially reduced, suggesting that uniparental inheritance assures the stability of the mitochondrial genotype. Unlike their homoplasmic counterpart, NZB-129 heteroplasmic mice showed altered behavior, reduced activity, accentuated stress response, and cognitive impairment [25].

In a human study conducted to address the consequences of mtDNA carryover, nuclear transfer between oocytes from women with different mitochondrial haplotypes was performed and the reconstructed oocytes were subjected to parthenogenesis and stem cell derivation (Figure 2) [24]. In the preimplantation embryos (*n* = 75), mtDNA heteroplasmy showed an average of 0.33%, and always less than 3%. In addition, seven of the eight ESC lines showed that nuclear transfer replaced mtDNA completely below the detection level for up to 32 passages over eight months. Conversely, in the MR-PS12 stem cell line, whose mtDNA is composed of haplotype L3 from the cytoplasm donor and haplotype H1 from the nuclear genome donor, the mtDNA heteroplasmy of the mtDNA H1 haplotype increased after genome transfer, from 1.3% at derivation to 53.2% at passage 36 (mtDNA stochastic drift) (Table 1). In addition, clonal expansion from single cells at passages 18, 30, 36, 40, 50, and 60 resulted in colonies with diverse mtDNA heteroplasmy, ranging from 0–90% of the H1 haplotype. Furthermore, two of three diploid ESC lines derived using somatic cell nuclear transfer (NT-ESCs), whose mtDNA haplotype combination between donor and recipient was the same (a somatic cell of the K1 haplotype and an oocyte of the L0 haplotype), showed rapid mtDNA drift between passages 0 and 10, and reached homoplasmy for the somatic mtDNA genotype between passages 15 and 25 (mtDNA directional shift) [21]. Somatic cell nuclear transfer can replace mitochondria in a similar way to genome transfer between oocytes. Other groups also reported that several isolated ESC lines after MST or PNT between healthy donors, or healthy donor and carriers with mutant mtDNA, demonstrated gradual loss of donor mtDNA and reversal to the maternal haplotype [16,22]. Approximately 20.5% of ESC lines (8 of 39) have shown an mtDNA reversion to that of the nuclear donor (Table 1) [16,19,22,24]. These results suggest that even a small amount of mtDNA carryover can affect the stability of the mtDNA genotype and consequently impair the effectiveness of the MR.

## 5. Mechanistic Insights for mtDNA Replication Bias

In two ESC lines using oocytes from women carrying pathogenic mutant mtDNA, there was a reversion to pathogenic mutants, whose mtDNA was from nuclear donors [22]. Although the clinical relevance of this fact is yet unclear, there are concerns regarding mtDNA reversion used for therapeutic applications of mitochondrial replacement. Therefore, further studies on the mechanism are required. Several causes of mitochondrial genetic drift have been proposed. First, it has been suggested that mitochondrial haplotypes and mitochondrial function may influence cell proliferation or mitochondrial genotype. However, cell competition assays between cells with the K1 haplotype and those with the L0 haplotype demonstrated that mitochondrial haplotype did not alter cell proliferation (Table 1) [24]. In addition, mitochondrial function in ESC lines and fibroblasts differentiated from ESC lines was not impaired by mitochondrial genotypes that differed from the maternal genome. These results suggest that mtDNA genetic drift is independent of mtDNA haplotype or mitochondrial function.

Second, some polymorphisms in the mtDNA conserved sequence box II (CSBII) containing sequences involved in the generation of mtDNA replication primer are suggested to be the cause of preferential replication of specific mtDNA haplotypes [22]. mtDNA transcription generates polycistronic mRNAs, which are processed to produce mature mRNAs, rRNAs, and tRNAs. Transcription from the heavy-strand promoter generates a truncated transcript that encodes for 12S rRNA, 16S rRNA, and two tRNAs. The light strand is transcribed by a single mitochondrial RNA polymerase to produce a primer for mtDNA replication. Transcription of the light strand can terminate prematurely within the non-coding region at a series of CSBs (I, II, and III) [26]. In the case of two of three reverted ESC lines (CSBII haplotype G6AG8 from the nuclear donor and G5AG8 from the cytoplasmic donor), the CSBII G6AG8 variant from the nuclear recipient’s mtDNA was claimed to have an advantage in mtDNA replication over the G5AG8 variant present in the donor’s mtDNA. In response to these results, Hudson et al. [27] analyzed the mtDNA sequence data published in Kang et al. [22] and showed that one of three ESC lines harboring identical donor-recipient combinations of CSBII variants did not revert to the nuclear genotype. A similar phenomenon was observed in other CSBII haplotype combinations including G6AG7-G6AG7, G6AG7-G6AG8, G5AG8-G6AG8, whose non-reverted:reverted ratios were 3:1, 6:0, and 1:2, respectively. These results imply that even if the same combination of CSBII polymorphisms is used, some ESC lines may revert while others may not revert at all [27]. Although the relevance of whether a reduction in the number of guanosine residues in the CSBII sequence is associated with reduced mtDNA replication [28], the exact mechanism by which these polymorphisms affect mtDNA replication remains unclear. Furthermore, there is insufficient evidence to support an advantage of matching the donor–recipient pairs based on CSBII haplotype similarity, since the incidence of reversion is similar between matched and non-matched CSBII haplotypes.

## 6. mtDNA–nDNA Mismatch

Mitochondrial function is under the control of both nuclear and mitochondrial genomes. After nuclear genome transfer, a different donor mtDNA is combined with the patient’s nDNA. Combining nDNA with donor-derived mtDNA raises concerns regarding cognitive behavior, macrosteatosis, inflammation, fibrosis, and development regarding mtDNA–nDNA compatibility in the reconstructed oocyte after genome replacement.

Some studies using animal models suggested a detrimental effect on development due to mtDNA–nDNA incompatibility. Fetterman et al. generated mitochondrial-nuclear exchange (MNX) mice using nuclear transfer, specifically C57BL/6J nuclear genome and C3H/HeN mtDNA [29]. MNX mice developed more macrosteatosis, inflammation, and fibrosis compared with the mice containing the C3H/HeN nuclear genome and fed an atherogenic diet [30]. Ma et al. [31] performed reciprocal genome transfer using PNT in zygotes from B6 and PWD mouse strains. Cybrid embryos (B6nDNA–PWDmtDNA) showed normal live birth rates (4%; 14 of 341) and F1 offspring with reduced male fertility, but reproductive fitness in females was unaffected. Conversely, the reciprocal combination (PWDnDNA–B6mtDNA) produced high embryonic loss and a live birth rate of 0.3% (1 of 359), resulting in post-implantation lethality in the F2 generation [31]. A study by Latorre-Pellicer et al. in conplastic animals, which are developed by backcrossing the nuclear genome from one inbred strain into the cytoplasm of another, showed that BL/6-mitNZB had a 16% longer lifespan, shorter telomeres, and lower tumor formation rate than those found in BL/6-mitC57 [32]. Although it is suggested that nDNA may not be compatible with mtDNA, highly inbred laboratory animals were used in these studies, which differ significantly from human populations due to diverse genetic backgrounds; therefore, these results should be interpreted with caution. Given these results, there is still not enough evidence to support the benefits of matching patients with a common mitochondrial genotype for nuclear transfer.

## 7. Mitochondrial Genome Alterations

Since a mitochondrial disease usually occurs when the pathogenic mtDNA heteroplasmy rate exceeds a given threshold in the same cell, it is possible to treat the disease by reducing or editing the mutant mtDNA. mtDNA genome editing techniques have been studied for many years.

As an example of mitochondrial genome editing in oocytes, Reddy et al. adopted the mitochondrially targeted transcription activator-like effector nucleases (mitoTALEN) approach using NZB/BALB heteroplasmic mice, which contained two mtDNA haplotypes. Microinjection of either a mitochondrial-targeted restriction endonuclease or mitoTALEN into oocytes from NZB/BALB heteroplasmic mice caused an mtDNA heteroplasmy shift, preventing germline transmission to the F1 generation [33].

Mitochondrially targeted zinc-finger nucleases (mtZFN) are reported to selectively degrade pathogenic mitochondrial genomes bearing large-scale deletions or point mutations [34]. Gammage et al. performed in vivo mtDNA genome editing using the mitochondrial DNA nuclease Fokl [35]. Mitochondrial genome editing was attempted using a mouse model that recapitulates the molecular features of mtDNA disease in cardiac tissue, the m.5024C > T tRNA^Ala^ mouse. mtZFNs were administered to individual mice using adeno-associated virus. As a result, an increase in the amount of pyruvate, a product of oxidative phosphorylation, was observed, suggesting an improvement in the function of mitochondrial oxidative phosphorylation due to elimination of mutated mtDNA [35]. On the other hand, there are concerns with this approach. For example, although double-strand breaks (DSBs) produced by the mtDNA nuclease Fokl lead to the degradation of mutated mtDNA, it is feared that removal of mutated mtDNA could result in a detrimentally low level of mtDNA copy number when the mutation load is very high.

In large part due its simplicity and accuracy, the CRISPR-Cas9 system is used for basic research and in clinical applications. DSBs introduced by CRISPR-Cas9 are repaired by non-homologous end joining or homologous recombination repair, which are inherent cellular mechanisms. However, some drawbacks such as high off-target effects, mosaicism, transmission of mutations to the next generation, etc., have been pointed out regarding the use of CRISPR-Cas9 for genome editing of early embryos. In addition, unrepaired DSBs persist and can result in frequent chromosome loss [36]. Therefore, genome editing using CRISPR for human germ lineages remains controversial.

The effectiveness of genome editing techniques for mtDNA may be limited because of the lack of mtDNA replication from the oocyte stage to early embryonic development. However, mtDNA genome editing technology harbors significant potential as a future substitute for MST and PNT because it does not require the use of donor-derived oocytes [37].

Recently, a new mtDNA precise editing technique was developed. RNA-free DddA-derived cytosine base editors (DdCBEs) catalyze C•G-to-T•A conversions in human mtDNA with high target specificity and product purity by fusing the bacterial intertoxin DddAtox with a transcriptional activator-like effector array protein and a uracil glycosylase inhibitor [38]. DdCBEs specifically convert cytosine to uracil within dsDNA and the enzyme targets double-stranded DNA in a mutagenic manner that relies on uracil DNA glycosylase to initiate base excision repair via uracil removal. This makes it particularly suitable for editing the mitochondrial genome, which lacks an efficient mechanism to repair DNA DSBs. When DdCBEs were applied to model disease-associated mtDNA mutations in human cells, alterations in the respiration rate and oxidative phosphorylation resulted. Although further studies are needed to fully elucidate the principles governing the efficiency and specificity of DdCBEs, the CRISPR-free DdCBE technique allows precise manipulation of mtDNA rather than removal of mtDNA copies. This has broad implications for elucidating the biological significance of mitochondria in early embryonic development, as well as the etiology and potential treatment of mitochondrial diseases.

## 8. Mitochondrial Replacement for Reproduction

Genome transfer for mitochondrial replacement is expected to have applications in the reproductive field as well. ATP produced by oxidative phosphorylation plays an important role in several biological phenomena including ovulation, fertilization, early embryonic development, and implantation. Although oxidative phosphorylation in mitochondria uses pyruvate as a substrate, glycolytic activity is restricted in fertilized oocytes because they show a low expression of phosphofructokinase. Consequently, embryonic metabolism is activated by a switch in energy source preference, from pyruvate obtained from cumulus cells in the oocyte oviduct fluid during the early cleavage stages to glucose during compaction and blastocele formation [39,40,41].

Primordial germ cells (PGCs), the primary undifferentiated stem cells which differentiate towards gametes (sperm or oocytes), originate from the early postimplantation epiblast cells and migrate to the developing genital ridge. At this stage, human PGCs contain around 200 copies of mtDNA (mean: 275.9, *n* = 24) [42] and maintain relatively fewer numbers during the early stages of oogenesis. Subsequently, the mtDNA copy number increases more than a thousand-fold during the process of differentiation from PGCs to mature oocytes (mean: 311.1 × 10^3^, *n* = 5) [19]. Since mtDNA is not replicated during preimplantation development, the mtDNA copy number per blastomere decreases with cleavage. In a mouse study, the median number of mtDNA molecules in mature oocytes was 249.4 × 10^3^ (*n* = 22), and the total number of mtDNA molecules within the entire preimplantation embryo remained constant (16–32 cell stage mean: 286.1 × 10^3^, *n* = 12; blastocyst mean: 280.8 × 10^3^, *n* = 15). Accordingly, the mtDNA copy number in blastomeres at the 16–32 cell stage decreased to a mean of 18.4 × 10^3^ (*n* = 11) [43], suggesting that mtDNA replication is not required for healthy preimplantation development. These two mtDNA bottleneck effects are thought to alter mtDNA heteroplasmy between generations.

The mtDNA copy number in blastocysts derived from older women (age: 39.8 years, range: 38–42 years, *n* = 154) is predominantly higher than that in blastocysts derived from younger women (age: 34.8 years, range: 26–37 years, *n* = 148) [44]. Nevertheless, the mtDNA copy number is known to be reduced in oocytes in older women, the opposite phenomenon of that observed in blastocysts. To explain this, the “quiet embryo hypothesis” was propounded by Leese [45]. As a result of less stress and lower metabolism in better quality embryos, the mtDNA copy number in high quality blastocysts is lower than that in impaired blastocysts. Conversely, oocytes under stress and with a reduced developmental potential tend to be more metabolically active, resulting in a compensatory increase in the number of mtDNA [44]. These findings are being applied to an ongoing clinical study [46] that uses mtDNA copy number as an embryo biomarker for embryo transfer.

Among all fertilization treatments, IVF shows the highest pregnancy rate, but even with IVF, the pregnancy rate decreases with age. Oocytes donated from women in their 20s substantially improves pregnancy rates, even if these fertilized oocytes are transferred into patients in their 40s, suggesting that the decline in pregnancy rates with age is largely due to a decline in egg function (oocyte aging). Most chromosomal abnormalities are known to occur during the first meiosis, and their underlying cause is thought to be an age-related decline in the oocyte cytoplasm. Several mechanisms have been considered to explain oocyte aging such as spindle dysfunction [47], early sister chromatid segregation by cohesin deterioration [48], telomere shortening [49], decreased cytoplasmic factor, and mitochondrial dysfunction.

The role of mitochondria in egg aging has received much attention in recent years. Oocytes from aged mice (>11 months) showed aggregated mitochondria more frequently than those from young mice (8–14 weeks) [50]. It is well-known that aged oocytes have a decreased mtDNA copy number and more mtDNA mutations, resulting in a decreased intracellular ATP content [51]. A 4977-bp deletion in mtDNA has been reported more often in oocytes from women older than 35 years than in women younger than 35 years [52]. When mitochondrial function in human oocytes was inhibited by the oxidative phosphorylation uncoupler trifluoromethoxy carbonyl cyanide phenylhydrazone (FCCP) supplementation in the culture media, substantially fewer meiotic spindles were detected in the FCCP-treated group (6 of 26 oocytes; 23.1%), compared to the control group (60 of 117 oocytes; 51.3%). This suggests that abnormalities in mitochondrial function with aging may impair spindle formation and cause abnormalities in chromosome disjunction [53].

Nuclear transplantation techniques are of interest not only for preventing the inheritance of mitochondrial disease, but for reproductive purposes, and several examples of fertility treatments using mitochondrial replacement and aging oocytes have been reported. In 1997, a live birth by ooplasmic transfer was reported, in which the cytoplasm from a healthy 27-year-old third party was injected into the eggs of a 30-year-old patient who had been infertile for 6.5 years and had failed to conceive after four embryo transfers [54]. Although cytoplasmic transplantation has shown some success in improving infertility (reviewed in [55]), concerns remain regarding the possibility of mixed mtDNA from the cytoplasmic donor and the biological mother. Due to limited evidence and the ethical issues, the FDA since banned cytoplasmic transplants.

Experiments using aged oocytes in mice showed that nuclear transfer of genomes from mice aged 10–12 months into the cytoplasm of young mice aged 3–5 months improved development to term from 6.3% in reproductively-aged oocytes to 27.1% in reconstructed oocytes [56]. In experiments using oocytes aged after ovulation by culturing them in vitro for 20 h after egg retrieval, nuclei that were transferred from the in vitro aged oocytes into young oocyte cytoplasm improved the blastocyst development rate, resulting live births [47]. Furthermore, cases of clinical application in humans have been reported. In 2017, a PNT was performed on a 34-year-old woman who was infertile for 15 years; the procedure resulted in the woman finally giving birth to a baby [57]. In 2019, MST was performed on nine women under 40 years of age who had several unsuccessful IVF attempts due to embryonic developmental arrest; MST resulted in two pregnancies and one live birth [58]. However, nuclear transfer is still controversial due to insufficient evidence regarding its efficacy and safety for reproduction purposes.

## 9. Conclusions

Great advances in research on mitochondrial diseases and genome transfer have been made in the last few years. However, there are some drawbacks associated with these treatments for preventing mutant mtDNA transmission. To ensure the clinically safe application of MRT, genome transfer techniques must be improved to reduce the amount of mtDNA carryover, together with a better understanding of mitochondrial biology, the mechanisms of mtDNA genetic drift, and the selection of compatible donors that do not compete with the recipient’s mtDNA and nDNA, which would help to elucidate the etiology of mtDNA disease and expand the biological applications of genome transfer.

## Figures and Tables

**Table 1:** mtDNA haplotype of nuclear and mitochondrial donors in stem cell lines.

Stem Cell Lines	mtDNA Haplotype	mtDNA Heteroplasmy
	Nucleus:Cytoplasm	
ST-ES1	H56:H2a	no mtDNA shift
ST-ES2	H2a:H56	no mtDNA shift
ST-ES3	H2a:H56	no mtDNA shift
ST-ES4	H44a:H13a	no mtDNA shift
ST-ES5	H1b:U5a	no mtDNA shift
ST-ES6	H1b:U5a	no mtDNA shift
ST-ES7	U5a:H1b	mtDNA shift
ST-ES8	U5a:H1b	mtDNA shift
ST-ES9	U5a:V3	no mtDNA shift
ST-ES10	V3:U5a	no mtDNA shift
ST-ES11	Hae:D1f	no mtDNA shift
ST-ES12	Hae:D1f	no mtDNA shift
ST-ES13	Hae:D1f	no mtDNA shift
ST-ES14	D4a:A2g	no mtDNA shift
ST-ES15	A2g:D4a	no mtDNA shift
13513ST-ES	T2b:T2	no mtDNA shift
3243ST-ES1	H49:B2k	mtDNA shift
3243ST-ES2	H49:B2k	mtDNA shift
31PNT	K:U	no mtDNA shift
36PNT	H:H	mtDNA shift
45PNT	L0:H	no mtDNA shift
47PNT	J:U	no mtDNA shift
55PNT	H:K	no mtDNA shift
MR-PS1	HV:C	no mtDNA shift
MR-PS2	HV:C	no mtDNA shift
MR-PS3	C:HV	no mtDNA shift
MR-PS4	C:I	no mtDNA shift
MR-PS5	HV:J	no mtDNA shift
MR-PS6	A:L3	no mtDNA shift
MR-PS7	A:L3	no mtDNA shift
MR-PS8	L0:L3	no mtDNA shift
MR-PS9	L3:U	no mtDNA shift
MR-PS10	L3:U	no mtDNA shift
MR-PS11	L3:HV	no mtDNA shift
MR-PS12	HV:L3	mtDNA shift
NT5	K:L0	no mtDNA shift
NT6	K:L0	mtDNA shift
NT8	K:L0	mtDNA shift

The cell lines ST-ES1, ST-ES2, ST-ES3, ST-ES4, ST-ES5, ST-ES6, ST-ES7, ST-ES8, ST-ES9, ST-ES10, ST-ES11, ST-ES12, ST-ES13, ST-ES14, ST-ES15, 13513ST-ES, 3243ST-ES1, and 3243ST-ES2 are from ref. [22]. The cell lines 31PNT, 36PNT, 45PNT, 47PNT, and 55PNT are from ref. [16]. The cell lines MR-PS1, MR-PS2, MR-PS3, and MR-PS4 are from ref. [19]. The cell lines MR-PS5, MR-PS6, MR-PS7, MR-PS8, MR-PS9, MR-PS10, MR-PS11, and MR-PS12 are from ref. [24]. The cell lines NT5, NT6, and NT8 are from ref. [21]. Reversed cell lines are shown in red color. Nonreversed cell lines that contained the same mtDNA haplotype combination as the reversed cell lines are shown in blue color. The remaining nonreversed cell lines are shown in black color.
